# Self-Medication with Drotaverine among Patients with Common Abdominal Symptoms and Treatment Efficacy from the Perspectives of Patients and General Practitioners—An Observational, Retrospective, Cross-Sectional Study Using Real-World Data

**DOI:** 10.3390/jcm11113156

**Published:** 2022-06-01

**Authors:** Piotr Eder, Piotr Kowalski, Agnieszka Mastalerz-Migas, Barbara Skrzydlo-Radomanska, Wojciech Cichy, Katarzyna Proga

**Affiliations:** 1 Department of Gastroenterology, Dietetics and Internal Medicine, Heliodor Swiecicki University Hospital, Poznan University of Medical Sciences, Ul. Przybyszewskiego 49, 60-355 Poznan, Poland; 2 Department of Pharmaceutical Chemistry, Faculty of Pharmacy, Medical University of Gdansk, Al. Hallera 107, 80-416 Gdansk, Poland; piotr.kowalski@gumed.edu.pl; 3 Department of Family Medicine, Medical University of Wroclaw, Ul. Syrokomli 1, 51-141 Wroclaw, Poland; agnieszka.mastalerz-migas@umed.wroc.pl; 4 Department and Clinic of Gastroenterology with the Endoscopy Laboratory of the Medical University of Lublin, Aleje Raclawickie 1, 20-059 Lublin, Poland; barbara.skrzydlo-radomanska@umlub.pl; 5 Faculty of Health Sciences, Calisia University-Kalisz, Ul. Nowy Swiat 4, 62-800 Kalisz, Poland; wbcichy@wp.pl; 6 PEX PharmaSequence Sp. z o.o., Ul. Klobucka 23, 02-699 Warszawa, Poland; katarzyna.proga@pexps.pl

**Keywords:** abdominal cramps, abdominal pain, antispasmodics, drotaverine, real-world data

## Abstract

In Poland, drotaverine is the most frequently purchased antispasmodic, yet there is a paucity of real-world data on its use. We evaluated the profiles of patients who used drotaverine, and we investigated prescription patterns among general practitioners (GPs). In this cross-sectional, questionnaire-based study, we asked patients who purchased drotaverine about their reasons for using it, its perceived efficacy, satisfaction with treatment, and physician consultation. We also asked GPs about the status of drotaverine in their practice. Among 650 recruited patients, 74% used drotaverine for pain, 67% for cramps, and 19% for abdominal discomfort. Approximately 83% of patients purchased drotaverine without a physician’s advice. Patients who used it after a physician’s advice were more frequently female, older, and less educated. For all symptoms, mean severity scores decreased by ~5 points (0–10 scale) after the first dose. Ninety-eight percent of patients were satisfied with drotaverine. Among 210 GPs, the percentages prescribing drotaverine were: 42% for irritable bowel syndrome, 89% for cholelithiasis, 60% as supportive therapy for urinary infections, 89% for nephrolithiasis, and 75% for menstruation pain. The GPs perceived drotaverine as more useful, effective, and tolerable than other drugs for abdominal pain or cramps. Drotaverine significantly reduced the severity of all symptoms for which it was taken, and it was perceived as effective and tolerable.

## 1. Introduction

Abdominal symptoms, such as pain, cramps, or discomfort, occur in up to a half of the general population [1,2]. Moreover, in general practice, about 1 in 10 patients report abdominal symptoms [3]. Various common abdominal symptoms, e.g., menstrual pain, renal colic, biliary colic, or spasms in the genitourinary tracts, are ascribed to spasms of smooth muscles. Therefore, antispasmodics are often used for pain relief in such conditions because these medications are safe and often available over the counter. The most commonly used antispasmodics include drotaverine, scopolamine, mebeverine, papaverine, and hyoscine. Despite different mechanisms of pharmacological action, these agents seem to have similar efficacy and safety in various abdominal complaints, but different medications seem to be preferred in different countries. 

Drotaverine is among the most frequently used antispasmodics in Poland. The safety and efficacy of drotaverine have been validated in many controlled trials in patients with recurrent abdominal pain, gastritis, irritable bowel syndrome, menstrual pain, or renal colic [4,5,6,7,8]. In clinical practice, however, drotaverine is used on a broader spectrum of patients. Unfortunately, no data have been published on the real-world use of drotaverine. Real-world studies play an important role in the lifecycle of non-prescription drugs, such as drotaverine. Such studies can provide evidence on whether medications are used in accordance with approved indications. Moreover, real-world studies provide important post-marketing safety data [9]. Therefore, we analyzed the profiles of patients who used drotaverine, we assessed the most common reasons for its use, and evaluated the effectiveness and tolerability of the drug. This questionnaire-based study was carried out among patients who purchased drotaverine in pharmacies and among general practitioners (GPs) who often recommend drotaverine to patients with abdominal symptoms. Our research is the first analysis of this kind carried out in Central and Eastern Europe, and one of the largest studies related to the discussed topic.

## 2. Patients and Methods

### 2.1. Study Design

This observational, retrospective, cross-sectional study using real-world data was carried out from September 2019 to February 2020 at pharmacies, among patients who purchased drotaverine and GPs who often prescribe the drug. We included patients who purchased drotaverine preparations (No-Spa, No-Spa Max, No-Spa Forte, No-Spa Comfort) within the month before inclusion. We chose the No-Spa brand, the most commonly used drotaverine preparation in Poland.

We recruited 97 pharmacists who worked in retail pharmacies in voivodship cities. Then, the pharmacists invited adult (18 years or older) patients who bought any No-Spa preparation. The No-Spa purchase had to be unsolicited by the pharmacist. Pregnant women were not included. Each pharmacist recruited 10 patients; those who signed informed consent to participate in the study were contacted by phone to complete a questionnaire. Patients answered questions about the reasons for using drotaverine (pain, cramps, abdominal discomfort), the perceived intensity of symptoms before and after the first dose (semiquantitative scale, 0–10: 0 = no pain, 10 = most severe pain), satisfaction with treatment (very satisfied, satisfied, rather satisfied, dissatisfied), the perceived time of onset (<15 min, 16–30 min, 31–60 min, and >60 min; very fast, rather fast, rather slow, and slow), and duration of symptom relief since the first dose (<4 h, <8 h, <12 h, <24 h, symptoms never returned). Moreover, we asked whether the patients purchased drotaverine after a physician’s advice. 

The recruitment of GPs was done with random quota sampling to obtain a representative sample for the type of residency across voivodeships. The eligible subjects were GPs with 3 or more years of experience in general medicine, who consulted 400 or more patients monthly and prescribed No-Spa preparations. All the physicians contacted during the recruitment satisfied the criteria of a minimum number of consulted patients and No-Spa prescription. The GPs filled out an online questionnaire on how they approached the patients with abdominal symptoms and on their views on different medications they prescribed for these symptoms. 

The study did not direct any additional diagnostic or therapeutic interventions, additional specialist consultations, or medical visits. Consequently, no formal ethical approval was required. The study was conducted in accordance with the principles of the International Code of Ethics for Market Research ESOMAR and the law in force in Poland (Act on Pharmaceutical Law, Act on Personal Data Protection, Act on the Medical Profession) and in compliance with Sanofi’s procedures for monitoring and reporting safety information of No-Spa, No-Spa Max, No-Spa Comfort, and No-Spa Forte. These procedures were carried out by PEX Pharma Sequence on behalf of Sanofi. Informed consent was obtained from all subjects involved in the study.

### 2.2. Statistical Analysis

Data were presented with descriptive statistics. The Kruskal–Wallis test, the Wilcoxon test, or the Friedman test was used for continuous or ordinal variables to assess the statistical significance of subgroup differences or variable differences. Categorical variables were compared using the chi-square test. A significance level value of *p* < 0.05 (confidence level 0.95) was adopted when defining statistical significance. The analyses were carried out using SPSS 24 software.

## 3. Results

### 3.1. Survey among Patients

We included 650 patients (women, 88%) with a mean age of 43 ± 14 years (range, 18–89 years). Most patients (58%) had normal weight (BMI, 18.5–24.9), whereas 4% were underweight (<18.5), 29% were overweight (25–29.9), and 9% were obese (>30). Most patients had tertiary education (57%), followed by secondary education (28%), post-secondary education (9%), vocational education (5%), and lower secondary or primary education (2%) (Supplementary Table S1). Sixty-five percent of patients purchased drotaverine within a week of enrolment. 

Ninety-one percent of all patients re-purchased drotaverine, whereas 9% purchased it for the first time. Among the patients purchasing the drug for the first time, drug information was commonly sourced from pharmacists (43%), friends or relatives (35%), and physicians (20%). The patients who re-purchased drotaverine received information primarily from family and friends (44%), pharmacists (30%), physicians (26%), and TV advertisements (28%). 

**Figure 1:**
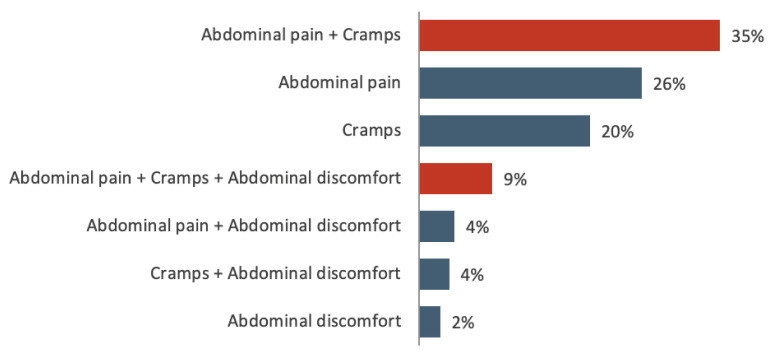
Proportions of patients reporting abdominal pain, cramps, or discomfort.

In the entire sample, 74% of patients used drotaverine for abdominal pain (±other symptoms), 67% for cramps (±other symptoms), and 19% for abdominal discomfort (±other symptoms). Most patients (54%) reported more than one symptom (see Figure 1 for details). Overall, 42% of the patients had menstrual pain, and 30% had abdominal pain unrelated to menstruation. The perceived impact of symptoms on daily activities was strong in 29% of patients, while 40%, 16%, and 15% reported moderate, small, and no impact, respectively (Supplementary Table S1). 

**Figure 2:**
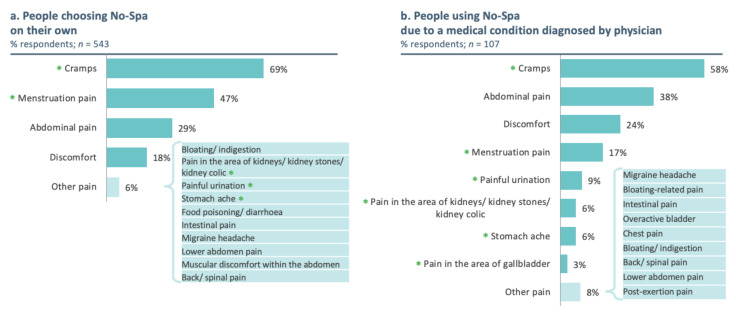
Symptoms prompting drotaverine purchase without (**a**) or with a physician’s advice (**b**). *p*-values for the chi-squared test comparing the frequency of symptoms between participants who purchased drotaverine with or without physician advice were <0.05 for cramps and <0.01 for the remaining symptoms (as indicated by * for the significant differences).

The majority of the patients (83%) purchased No-Spa without a physician’s advice. In these patients, the following symptoms were commonly observed: abdominal cramps (69%), menstrual pain (47%), and abdominal pain unrelated to menstruation (29%, Figure 2a). In the rest of the patient population (17%, *n* = 107), taking No-Spa after a physician’s advice, 58% reported abdominal cramps, 38% reported abdominal pain unrelated to menstruation, and 24% of them reported abdominal discomfort (Figure 2b).

Patients purchasing drotaverine upon a physician’s advice, compared to those who purchased it without such advice, were more frequently male (24% vs. 10% were men; *p* < 0.001), had tertiary education less frequently (45% vs. 59% with tertiary education; *p* = 0.004), and were older (50 vs. 42 years on average; *p* < 0.001). As the starting dose, 58% of patients took 80 mg of drotaverine, 30% took 40 mg, and 11% took 160 mg. The dose of 80 mg was taken more frequently by patients without a physician’s advice (60% vs. 49%, *p* = 0.028), whereas 160 mg of drotaverine was often taken after a physician’s advice (19% vs. 9%, *p* = 0.004). Furthermore, 30% of the patients took a 40 mg dose, equally in both patient populations, with or without a physician’s advice.

The perceived onset of action was <15 min for 8% of patients, 16–30 min for 53%, 31–60 min for 32%, and >60 min for 8% of the patients. The onset of action was similar for all doses (data not shown). Fifteen percent of patients regarded the onset of action as very fast, 78% as rather fast, 5% as rather slow, and 1% as slow. The duration of symptom relief was reported to be <4 h for 26% of patients, <8 h for 24%, <12 h for 16%, and <24 h for 9%. Notably, the symptoms never returned after the first dose of drotaverine in 25% of patients. 

**Figure 3:**
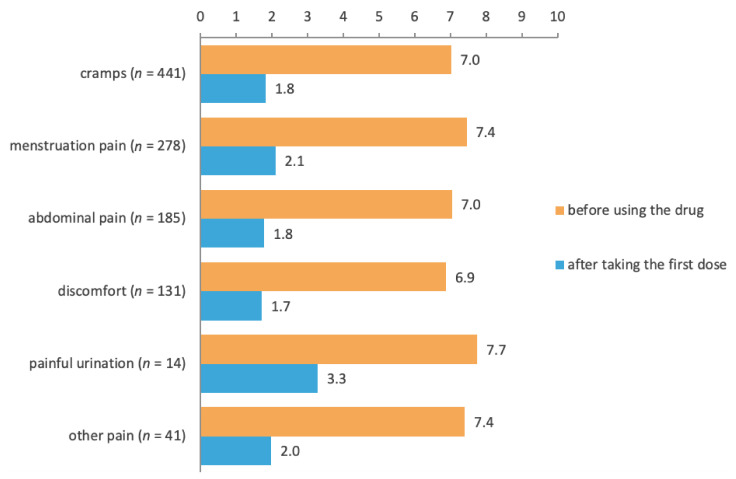
Subjective severity of symptoms before and after taking the first dose of No-Spa by symptoms. *p*-values from the Wilcoxon test comparing symptom severity before and after drotaverine were <0.01 for all symptoms.

Drotaverine was similarly effective for all symptoms: after the first dose, the mean severity scores had decreased by ~5 points for cramps, menstruation pain, abdominal pain unrelated to menstruation, and discomfort (in most cases caused by pain and/or cramps) (Figure 3). Moreover, the mean severity scores similarly decreased (~5 points) for all doses (40 mg, 80 mg, 160 mg) and in patients with or without a physician’s advice (data not shown).

The overall pharmacological effect of drotaverine was excellent in 31% of patients, good in 55%, sufficient in 13%, followed by not good or poor (1% each). The effect was similar for individual symptoms (data not shown). Taken together, 42% of the patients were very satisfied with the treatment, 56% were satisfied, 1% were rather dissatisfied, and 1% were dissatisfied. The impact of drotaverine on daily activities was largely positive (62%, positive; 9%, rather positive; 29%, absent; and <1%, negative or rather negative). The impact on physical activity was reported as follows: positive (50%), rather positive (8%), absent (42%), or negative or rather negative (<1%). The impact on emotional condition was positive in 29% of patients, 6% experienced a rather positive impact, whereas 64% and <1% of patients had either no impact or a negative/rather negative impact, respectively.

### 3.2. Survey among General Practitioners

We enrolled 210 GPs (51% male) with a mean experience in general medicine of 21 years. The GPs declared that 17% of patients in their practices had abdominal pain or cramps. Among these patients, GPs estimated that 39% had gastrointestinal diseases, 26% had urinary tract diseases, 23% had biliary tract diseases, and 12% had menstruation pain. When GPs were asked to name the five most frequent indications to prescribe drotaverine, the most common were as follows: cholelithiasis (83% of GPs), nephrolithiasis (80%), menstruation pain (54%), urolithiasis (49%), bladder inflammation (43%), and irritable bowel syndrome (39%). 

The proportions of GPs who prescribed antispasmodics were as follows: 96% for irritable bowel syndrome (42% of GPs prescribed drotaverine), 41% as supportive therapy for gastric and duodenal ulcers (drotaverine, 39%), 97% for cholelithiasis (drotaverine, 89%), 64% as supportive therapy for urinary infections (drotaverine, 60%), 98% for nephrolithiasis (drotaverine, 89%), and 83% for menstruation pain (drotaverine, 75%).

For the 40 mg dose, the perceived onset of action was up to 15 min for 37% of GPs, 16–30 min for 41%, 31–60 min for 22%, and >60 min for <1%. GPs regarded the onset of action for this dose as very fast (50%) or moderate (50%). On the other hand, for the 80 mg dose, the perceived onset of action was up to 15 min for 47% of GPs, 16–30 min for 38%, and 31–60 min for 15%. Moreover, none of the GPs perceived the onset of action to be longer than 60 min; 62% of them regarded the onset as very fast and 38% as moderate. None of the GPs deemed the onset with any of the doses (40 and 80 mg) to be slow or very slow.

**Figure 4:**
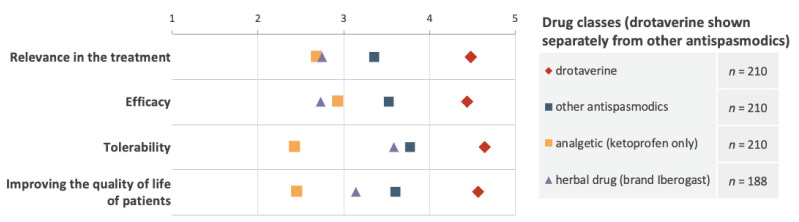
Subjective scores of drotaverine and other medications concerning the usefulness in daily practice, efficacy, tolerability, and effect on the quality of life (higher scores indicated greater relevance, efficacy, tolerability, and quality of life improvement).

In treating patients with abdominal pain or cramps, GPs perceived drotaverine to be more useful in daily practice. When compared with other antispasmodics (hyoscine, mebeverine, alverine), ketoprofen, and herbal preparations, drotaverine was more effective, more tolerable, and had a greater impact on quality of life (Figure 4).

## 4. Discussion

In this study, drotaverine was primarily employed to tackle abdominal pain or cramps that substantially impacted daily activities based on real-world data. The most common cause of the pain was menstruation. Over 80% of all patients in our sample purchased drotaverine without a physician’s advice. Drotaverine was the most prescribed antispasmodic for nearly all indications in general practices in Poland. Drotaverine significantly reduced the intensity of all symptoms and decreased the burden of symptoms on daily activities. The overall treatment satisfaction with drotaverine was high among patients. Similarly, GPs found drotaverine to be more effective and tolerable than other antispasmodics or ketoprofen in patients with abdominal pain or cramps. 

Drotaverine is available in Poland without prescription, and it is therefore not surprising that most patients purchased drotaverine without a physician’s advice. Self-medication is an increasing trend worldwide, and many people tend not to consult a physician when experiencing symptoms [10]. Instead, they might consult a pharmacist or other professional medical staff to get immediate help. In Europe, the proportion of people using non-prescribed medicines varies between countries: <20% in Italy, >50% in Poland, and 70% in Finland [11]. The most commonly used non-prescribed drugs include non-steroidal anti-inflammatory drugs, gastrointestinal drugs, and cough remedies [12]. In previous studies, self-medication was noticed to be associated with education status, family factors, social factors, legal regulations, drug availability, and exposure to advertisements [10,13]. In our study, patients who purchased drotaverine without a physician’s advice were younger, better educated, and more frequently female than those who obtained advice. In line with our study, the use of over-the-counter drugs in the EU was greatest among women and those who completed tertiary education [11]. We suppose that the frequent use of drotaverine in young women can be partly due to irritable bowel syndrome and menstrual pain [14]. Moreover, in Poland, women receive tertiary education more often than men do, which could be responsible for the high use of drotaverine without a physician’s advice among women. A higher frequency of drug use among women than men could be because women perceive their health status as worse than men [15]. In our study, nearly 90% of all patients were women. Moreover, due to traditionally ascribed gender roles, it might be easier for women to admit pain [16]. Similarly to our study, a Eurostat analysis found that the intake of non-prescribed medicines was highest among people aged 25–44 years (37% of all ages) [11]. The reason could be that older individuals, who often suffer from many chronic diseases, are more likely to see a physician and obtain a prescription. 

We observed that patients who purchased drotaverine without a physician’s advice took lower first doses. This observation can be associated with less serious symptoms that do not prompt a consultation with a physician. Moreover, patients who take drugs without a physician’s advice might not use higher doses because of the fear of adverse effects. 

Drotaverine reduced the severity of all surveyed symptoms, and the overall effect of drotaverine was assessed to be excellent or good by nearly 90% of patients. We observed that drotaverine was equally effective for all doses in patients irrespective of the drug being purchased with or without a physician’s advice. Nearly all patients were satisfied with the treatment, which lessened the burden of symptoms in daily activities. Over 90% of patients declared that the onset of action of drotaverine was fast or rather fast (similarly to all GPs). The high treatment satisfaction with drotaverine suggests that it can improve quality of life. Therefore, further studies should assess the impact of drotaverine on quality of life in primary care and among patients using drotaverine without a physician’s advice [17]. 

The GPs in our study estimated that about 20% of their patients reported abdominal symptoms. Similarly, in a prospective study encompassing ~3000 patients in general practice, 14% wished to consult because of abdominal complaints [18]. However, the estimates in our study were calculated based on the declarations of GPs and not by a systematic study. According to the GPs in our sample, the most common indications for drotaverine use were cholelithiasis, nephrolithiasis, and menstruation pain. Except for irritable bowel syndrome, drotaverine was the most prescribed antispasmodic. Controlled trials showed that drotaverine is more effective than both placebo and mebeverine in patients with irritable bowel syndrome [19,20]. Thus, concerning patients with irritable bowel syndrome in Poland, the reasons behind the preference of other antispasmodics over drotaverine are still unclear. However, overall, in patients with abdominal pain or cramps, GPs preferred drotaverine to other antispasmodics and ketoprofen, possibly due to drotaverine’s higher perceived efficacy and tolerance. In contrast, drotaverine was the third most popular antispasmodic among healthcare professionals in India, after camylofin and dicyclomine [21]. We observed that GPs preferred drotaverine to ketoprofen for menstrual pain. Non-steroidal anti-inflammatory drugs (NSAIDs), such as ketoprofen, seem more effective in women with menstrual pain [22,23]. However, GPs could recommend other NSAIDs, which we did not ask about in our questionnaire. 

The limitations of our study need to be mentioned. First, we based our analyses on declarative data, which can lead to recollection bias. Future studies could be improved by a prospective collection of data or an analysis of medical charts in general practices. However, subjective measures need to be used while analyzing the effect of treatments on pain and everyday activities. Lastly, the data were not gathered with validated questionnaires. However, the questions were easy to understand, and similar basic questionnaires are often used without the need for validation [24,25]. We provided data on the use of drotaverine within a real-world setting in a large and representative sample of patients and GPs, which is the strength of our study. To our knowledge, this work is the first to describe research on a single biologically active substance on such a significant number of participants.

In conclusion, employing real-world data, our study showed that drotaverine was used primarily without a physician’s advice to restrain symptoms, e.g., abdominal pain, menstrual pain, or abdominal cramps. Drotaverine significantly reduced the severity of all symptoms for which it was taken. It was also the most commonly prescribed antispasmodic in general practice in Poland. The drug was perceived as effective and tolerable by patients and GPs. The overall treatment satisfaction was discerned to be high, with no new safety signals.

## Data Availability

The data presented in this study are available on request from the corresponding author.

## Supplementary Materials

The following supporting information can be downloaded at: https://www.mdpi.com/article/10.3390/jcm11113156/s1, Table S1: Sample characteristics and impact of drotaverine.
