# Supplementary material for: Self-Medication with Drotaverine among Patients with Common Abdominal Symptoms and Treatment Efficacy from the Perspectives of Patients and General Practitioners—An Observational, Retrospective, Cross-Sectional Study Using Real-World Data
Source: J Clin Med. 2022 Jun 1;11(11):3156. doi: 10.3390/jcm11113156 (PMC9181793; doi:10.3390/jcm11113156)
Supplement: Supplementary file 1 [file jcm-11-03156-s001.zip › jcm-1700738-supplementary.pdf]

Supplemental Table S1. Sample characteristics and impact of drotaverine

	N=650
Mean age±standard deviation (years)	43±14
Women, %	88%
Body-mass index, %	
<18.5	4%
18.5–24.9	58%
25-29.9	29%
>30	9%
Education, %	
tertiary education	57%
secondary education	28%
post-secondary	9%
vocational	5%
lower secondary or primary	2%
Patients purchasing drotaverine without physician's advice, %	83%
Type of drotaverine purchase, %	
first-time	9%
re-purchase	91%
Sources of information on drotaverine among first-time purchasers, %	
pharmacists	43%
friends or relatives	35%
physicians	20%
TV advertisements	18%
a known drug (a healthcare professional)	3%
radio advertisements	2%
Sources of information on drotaverine among re-purchasers, %	
family and friends	44%
pharmacists	30%
physicians	26%
TV advertisements	28%
experience with the drug / brand used for a long time	6%
radio advertisements	2%
advertising (general)	1%
search engine / the internet	1%
cannot remember / hard to say	3%
Initial drotaverine dose, %	
40 mg	30%
80 mg	58%
160 mg	11%
other	1%
Perceived onset of action of drotaverine, %	
<15 min	8%
16-30 min	53%
31-60 min	32%
>60 min	8%
Perceived speed of action of drotaverine, %	
very fast	15%
rather fast	78%
rather slow	5%
slow	1%

Duration of symptom relief after drotaverine, %	
The symptoms disappeared completely after taking the first dose	25%
<4 h	26%
<8 h	24%
<12h	16%
<24 h	9%
Overall pharmacological effect of drotaverine, %	
excellent	31%
good	55%
sufficient	13%
not good	1%
poor	1%
Treatment satisfaction with drotaverine, %	
very satisfied	42%
satisfied	56%
rather dissatisfied	1%
dissatisfied	1%
Impact of drotaverine on daily activities, %	
positive	62%
rather positive	9%
absent	29%
negative or rather negative	<1%
Impact of drotaverine on physical activity, %	
positive	50%
rather positive	8%
absent	42%
negative or rather negative	<1%
Impact of drotaverine on emotional condition, %	
positive	29%
rather positive	6%
absent	64%
negative or rather negative	<1%
